# Proceedings of the 3rd BEAT-PCD Conference and 4th PCD Training School

**DOI:** 10.1186/s12919-018-0161-6

**Published:** 2018-12-18

**Authors:** Hannah Farley, Bruna Rubbo, Zuzanna Bukowy-Bieryllo, Mahmoud Fassad, Myrofora Goutaki, Katharine Harman, Claire Hogg, Claudia E. Kuehni, Susana Lopes, Kim G. Nielsen, Dominic P. Norris, Ana Reula, Nisreen Rumman, Amelia Shoemark, Hannah Wilkins, Agatha Wisse, Jane S. Lucas, June K. Marthin

**Affiliations:** 1 MRC Harwell Institute, Harwell Campus, Oxfordshire, UK; 2 Department of Physiology, Anatomy and Genetics, Parks Road, Oxford, Oxfordshire, UK; 3 Primary Ciliary Dyskinesia Centre, NIHR Southampton Biomedical Research Centre, University of Southampton and University Hospital Southampton NHS Foundation Trust, Southampton, UK; 4 Institute of Human Genetics, Polish Academy of Sciences, Poznan, Poland; 5 Genetics and Genomic Medicine Programme, University College London, UCL Great Ormond Street Institute of Child Health, London, UK; 6 Department of Human Genetics, Medical Research Institute, Alexandria University, 165 El-Horreya Avenue El- Hadra, Alexandria, 21561 Egypt; 7 Institute of Social and Preventive Medicine, University of Bern, Bern, Switzerland; 8 Paediatric Respiratory Medicine, University Children’s Hospital, University of Bern, Bern, Switzerland; 9 Royal Brompton Hospital, Sydney Street, London, UK; 10 Paediatric Respiratory Medicine, Imperial College, London, UK; 11 Department of Paediatrics, Royal Brompton Hospital, Sydney Street, London, UK; 12 CEDOC, Chronic Diseases Research Centre, NOVA Medical School, Faculdade de Ciências Médicas, Universidade Nova de Lisboa, 1169-056 Lisboa, Portugal; 13 Danish PCD & Child Centre, CF Centre Copenhagen, Paediatric Pulmonary Service, ERN Accredited, Department of Paediatrics and Adolescent Medicine, Copenhagen University Hospital, Rigshospitalet, Denmark; 14 Department of Physiology, University of Valencia, Valencia, Spain; 15 UCIM Departament, Instituto de Investigación Sanitaria Incliva, Valencia, Spain; 16 Department of Pediatrics, Makassed Hospital, East Jerusalem, Palestine; 17 Division of Molecular and Clinical Medicine, University of Dundee, Dundee, UK; 18 University Hospital Southampton NHS Foundation Trust, Southampton, UK

**Keywords:** Primary ciliary dyskinesia, Chronic respiratory disease, Multidisciplinary

## Abstract

Primary ciliary dyskinesia (PCD) is a chronic suppurative airways disease that is usually recessively inherited and has marked clinical phenotypic heterogeneity. Classic symptoms include neonatal respiratory distress, chronic rhinitis since early childhood, chronic otitis media, recurrent airway infections leading to bronchiectasis, chronic sinusitis, laterality defects with and without congenital heart disease including abnormal situs in approximately 50% of the cases, and male infertility. Lung function deteriorates progressively from childhood throughout life. ‘Better Experimental Approaches to Treat Primary Ciliary Dyskinesia’ (BEAT-PCD) is a network of scientists and clinicians coordinating research from basic science through to clinical care with the intention of developing treatments and diagnostics that lead to improved long-term outcomes for patients. BEAT-PCD activities are supported by EU funded COST Action (BM1407). The third BEAT-PCD conference and fourth PCD training school were held jointly in February 2018 in Lisbon, Portugal. Presentations and workshops focussed on advancing the knowledge and skills relating to PCD in: basic science, epidemiology, diagnostic testing, clinical management and clinical trials. The multidisciplinary conference provided an interactive platform for exchanging ideas through a program of lectures, poster presentations, breakout sessions and workshops. Three working groups met to plan consensus statements. Progress with BEAT-PCD projects was shared and new collaborations were fostered. In this report, we summarize the meeting, highlighting developments made during the meeting.

## Introduction

Primary Ciliary Dyskinesia (PCD) is a multi-organ genetic disease associated with bronchiectasis, chronic rhinosinusitis, male infertility and *situs inversus* (SI) [1]. The estimated incidence in Europe is generally 1:10,000 [2, 3], but it can be much more common in isolated or consanguineous populations, such as the Dutch Volendam, where the incidence is 1:400. In health, motile cilia in the respiratory tract clear pathogen-laden mucus from the airways. Impaired mucociliary clearance, caused by abnormal ciliary function, leads to persistent upper and lower respiratory tract symptoms starting at birth [4, 5] and with time leads to bronchiectasis [6] and impaired lung function [7]. Sperm flagella have a similar ultrastructure to cilia and most but not all men with PCD are infertile [8]. In the early embryo, motile nodal cilia control the establishment of left-right asymmetry; approximately half of PCD patients exhibit situs inversus totalis (SIT) while 6-12% have situs ambiguus (SA), which is often associated with complex congenital cardiac defects [4]. To date there are no specific treatments for PCD, and hence no evidence based guidelines for clinical management of patients.

‘Better Experimental Approaches to Treat Primary Ciliary Dyskinesia’ (BEAT-PCD; COST Action BM1407) is a network of scientists and clinicians coordinating research from basic science through to clinical care, with the intention of developing treatments and diagnostics to improve long-term outcomes for people with PCD (www.BEATPCD.org). Within the first 30 months we have united a multidisciplinary network of 257 participants from 27 countries to collaborate through training schools, visits between institutions and conferences [9, 10]. This report covers the proceedings of the 3^rd^ Annual BEAT-PCD Conference and 4^th^ Annual Training School, held in Lisbon in February 2018, which included state-of-the-art lectures, oral presentations, workshops, a trainee networking forum and a poster session.

Workshops at previous BEAT-PCD meetings had identified and prioritised research projects; for example, a multinational study of the natural variability of spirometry values (PROVALF-PCD), the effects of nutritional status and a review of experimental models of PCD. BEAT-PCD clinicians have written an expert statement for the management of children with PCD [1], and work groups (WGs) continue to develop consensus guidelines for specific aspects of diagnosis and management, such as diagnosis via transmission electron microscopy (TEM), infection control, a definition for pulmonary exacerbations and physiotherapy management. An active Training School within the BEAT-PCD network promotes education, training and career development to its 110 junior members. Interaction between senior academics, clinicians, students, post-doctoral fellows and invited speakers from academia and industry at conferences, workshops and Training Schools provides career development opportunities.

### Work group activities during the conference and training school

**Fig. 1:**
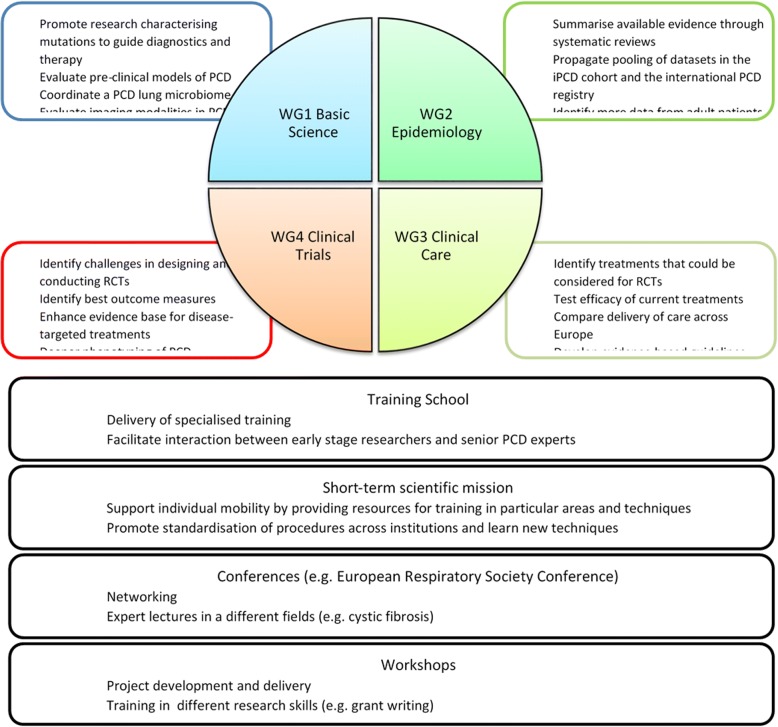
A diagrammatic representation of the aims of work groups within BEAT-PCD and COST action-associated activities. WG = work group

BEAT-PCD activities are coordinated by four collaborative WGs: basic science, epidemiology, clinical care and clinical trials (Fig. 1).

WG1 (basic science) aims include highlighting recent scientific advances that will translate into clinical research and ultimately clinical practice. Plenary talks addressed the concepts and mechanisms underlying left-right patterning in the early embryo; mucus composition and how it affects mucociliary transport; and potential small molecule therapies that can alter mucus composition and therefore may be able to alter mucus clearance. WG1 held workshops on the nature of laterality and on model organisms that can be used in PCD research.

WG2 (epidemiology) presented information from the international PCD (iPCD) cohort and PCD registry [11, 12]. The iPCD cohort grows exponentially and currently consists of 3507 patients from 20 countries and 26 centres. Several recent studies nested in the iPCD cohort were presented. The PCD registry is now part of the European Reference Network (ERN), more specifically the ERN-LUNG network. Countries outside the EU can also be part of the ERN-LUNG network and contribute with data on PCD patients. Another project within WG2, 3 and 4 is the development of a standardized PCD Proforma for patient follow-up and prospective data collection, as presented by Myrofora Goutaki.

WG3 (clinical care) ran workshops discussing consensus statements on infection prevention and control for PCD patients, and defining respiratory exacerbations (in partnership with WG4). A research study looking at the differences in delivery of care for PCD across Europe aims to investigate variations in models of service delivery for patients with suspected or confirmed diagnosis of PCD within and between European countries. The International PCD Physiotherapy Network (IPCDPN) presented plans to publish a consensus statement on “Effective physiotherapy” for PCD patients during the coming year.

WG4 focuses on developing clinical trials for PCD, with two currently underway [13–15]. Presentations were given on research that aims to improve outcome measures available in PCD trials. Age -appropriate quality of life measures, first developed in the BESTCILIA project (FP7) are now being further validated and translated by the BEATPCD network [16–18]. Workshops were held on biomedical imaging techniques as potential outcome measures in PCD.

### State-of-the-art lectures

Plenary lectures were given by keynote speakers on topics relevant to BEAT-PCD including left-right asymmetry, super-resolution imaging to support PCD diagnostics, and mucus disorders which might mimic PCD.

Three plenary lectures focussed on explaining the mechanisms underpinning left-right (L-R) patterning. Dominic Norris (UK) explained how the L-R axis is fundamental in visceral organ patterning and placement. He emphasised the importance of standardizing the nomenclature used to describe heterotaxia, for example avoiding the usage of situs solitus (SS) with isolated organ situs defects, e.g. situs solitus with dextrocardia should be more properly referred to as situs ambiguous or heterotaxy. Finally, he gave an overview of L-R establishment in human embryos. He focused 199 the heart is severely affected by L-R patterning defects, namely its highly asymmetric anatomy. The developmental genetic cascades key to forming this anatomy require clear and appropriate early left-right signalling, to enable their correct temporal and spatial activation [19].

Leonor Saúde (Portugal) elaborated on the history of our understanding of how left and right are first established in the embryo and on how this varies between different species. Some rely on cilia-driven flows (mice, rabbit, fish and Xenopus), while others utilise cell movements to convert symmetrical into left-right asymmetric gene expression patterns (e.g. chicken). Downstream of ciliary motility in the embryo, the *Nodal-Lefty-Pitx2* gene cascade is expressed on the left but not right side of the organism in the majority of bilaterians; organisms with chiral asymmetries, such as snails, also use this genetic cascade.

Adam Shapiro (Canada) spoke about laterality defects in patients with PCD from a clinical perspective. He defined the spectrum of situs abnormalities [20], explaining that congenital heart disease with heterotaxy is 200-fold higher in PCD patients than in the general population [21]. A recent prospective study found laterality defects in 55% of PCD cases [22]. Clinical outcome in patients with PCD and heterotaxy has been reported to be worse than in those with PCD alone, without heterotaxy [23]. To fully evaluate the presence of heterotaxy, abdominal ultrasound and echocardiography for laterality defects were advised to be included in the diagnostic work-up for PCD.

Following on from more conceptual presentations about super-resolution microscopy (SRM) at 2017’s BEAT-PCD Conference [9], Sharon Dell and Vito Menella (Canada) presented a study exploring the use of these techniques in PCD diagnosis. Three-dimensional structured illumination microscopy (SIM) quantification and stochastic optical resolution microscopy (STORM) imaging were evaluated in a two-arm study exploring whether diagnostic specificity could be improved. These techniques showed increased specificity compared to transmission electron microscopy (TEM) and immunofluorescence (IF). SRM analysis can be automated to reduce subjectivity and is faster than diagnosis using a combination of IF and TEM. STORM reveals the detailed structures of ciliary proteins and thus may be beneficial in difficult diagnostic cases.

David Thornton (UK) discussed the importance of mucus in physiological airway function and the clinical implications of mucus disorders. Gel-forming mucins (MUC5AC and MUC5B) make up the main structural components of the protective mucus barrier in the airways. In mouse lungs, MUC5B [24] expression predominates during homeostasis. *Muc5b* null mice show deficient mucociliary clearance and reduced survival compared to wild-type. MUC5AC has been found to be crucial for heterogeneous mucus plugging, a component of airway hyper-reactivity in asthma [25]. Inflammation increases the elasticity of mucus by stimulating the secretion of stored mucins, thereby increasing their relative concentration. Moreover, inflammatory signalling cascades lead to an increase in reactive oxygen species, which promote mucin cross-link formation [26] and further increase mucus elasticity. Strategies for tackling mucus obstruction of airways were introduced, including restoring mucus hydration and reducing mucin levels.

### Oral presentations

A number of short oral presentations on PCD research were given throughout the conference.

#### Basic sciences oral presentations

The scientific presentations primarily included reports of novel PCD causative genes identified within the last year.

Ewa Zietkiewicz (Poland) presented on the genetic screening of PCD mutations in the Polish/Slavic population. Classical and next generation sequencing in 377 families determined the population-specific profile of the three most frequently mutated PCD-causing genes (*SPAG1, CCDC39, CCDC40*); some of these mutations had not previously been described in European populations. The study also identified a novel candidate PCD gene, *C11orf70*. Nasal brushing samples from patients showed absence of cilia motility and lack of outer and inner dynein arms. Knocking down *c11orf70* in zebrafish resulted in defective ciliary motility. Protein truncating mutations found in the Polish cohort suggest that *MNS1* could be another PCD candidate gene.

Inga Höben (Germany) also reported on the role of *C11orf70* in PCD. She explained that *C11orf70* is expressed in nasal biopsies as well as differentiated primary human airway cell cultures, consistent with a role in motile ciliogenesis. Inga demonstrated that patients with *C11orf70* mutations exibit immotile cilia and sperm flagellae, and that these both lack inner and outer dynein arms, implicating C11ORF70 in cytoplasmic pre-assembly of dynein arms. Immunofluorescent staining of patient cilia showed reduction of dynein heavy chains, whilst no effect was seen on other ciliary structures (such as radial spokes and nexin links).

Hannah Farley (UK) presented analysis of ciliary motility in the trachea and embryonic node of *Pierce1*^*-/-*^ mice, which are known to exhibit situs defects [27]. By quantifying embryonic nodal ciliary motility, Hannah demonstrated that the laterality defects resulted from dyskinetic ciliary beating and disrupted flow in the embryonic node. She further described reduced beat frequency and aberrant beat pattern in tracheal cilia. TEM showed that tracheal cilia have a ultrastructural defects most similar to those seen in patients with *CCDC103* mutations, suggesting a role for PIERCE1 in the assembly of dynein arms. Motility analyses of sperm flagella in *Pierce1*^*-/-*^ mutant mice are ongoing.

Priyanka Anujan (UK) also focused on *Pierce1*, specifically on its role in motile ciliogenesis. *Pierce1* expression is upregulated during mucociliary differentiation of primary tracheal and nasal epithelial mouse cells. Enriched expression of *Pierce1* was observed in mouse organs containing motile cilia (lungs, testes, brain, ovary), along with other cilia motility genes (*Foxj1*, *Tekt1*). Studies of two zebrafish *pierce1* models delivered contradicting results: *pierce1* morphants showed severe situs abnormalities and curved body axis, typically associated with cilia defects, while a proportion of CRISPR-Cas9-induced mutants showed only mild situs abnormalities.

Sandra Cindric (Germany) reported homozygous *STK36* (serine-threonine kinase 36) loss-of-function mutations in a PCD patient with situs solitus (SS), relatively high nNO (240ppb) and a stiff ciliary beat pattern with reduced amplitude [28]. The gene has been previously shown to be involved in motile ciliogenesis and cilia orientation in Drosophila and the mammalian oviduct [29, 30], and *STK36* mutations lead to impaired cilia orientation in human epithelial cells. Immunofluorescence staining indicated that STK36 was essential for assembly of central pair components and that it interacted with radial spoke proteins, which were crucial for STK36 recruitment to the axoneme.

David Hoogewijs (Switzerland) reported on androglobin (ADGB), a recently identified oxygen-binding protein. ADGB is strongly conserved across evolution and its expression is specific to the testis, where it is implicated in postmeiotic spermatogenesis and is essential for male reproduction [31]. A proportion of *Adgb*-knockout mice developed signs of PCD (hydrocephalus after birth, mucus accumulation, and congenital heart defects). The congenital heart defects observed are not typical situs-related defects, but instead myocardial hypertrophy; other defects not associated with PCD, such as polycystic kidney disease (PKD), were also observed. This diverges from the typical phenotype of mice null for causative PCD genes (situs defects, hydrocephalus).

Mahmoud Fassad (UK) presented a targeted NGS (next generation sequencing) programme aimed at identifying causative PCD mutations. A cilia-specific, multi-gene panel has been established to analyse 180 PCD patient families, and has identified nearly 80% of the causative mutations. Mutation distribution showed a population-specific profile, with different modal genes involved in each ethnic group (European cohort: *DNAH5, DNAH11*; Arab cohort: *CCDC39*, *CCDC40*; South Asian cohort: *LRRC6, CCDC103*). Two novel PCD candidate genes (*C11orf70* and *DNAH9*) were identified in this study, and the effect of knocking these down in Paramecium was also presented.

Pedro Sampaio (Portugal) reported an innovative study to determine when left-right pattern is established in the zebrafish embryo. Using mechanical manipulation to inactivate the left-right organizer, he defined a temporal window during which a directional fluid flow in the organizer is required. He showed that development of thoracic visceral organs (e.g. heart) and abdominal visceral organs (e.g. liver) is initiated at the same time point, and is coordinated by the same signalling pathways (Nodal-Lefty-Pitx2 cassette), leading to similar proportions of visceral and abdominal organ laterality defects.

#### Clinical oral presentations

Florian Halbeisen (Switzerland) presented on the evolution of PCD diagnostic testing in Europe among 2822 patients of the iPCD cohort. The proportion of PCD patients tested with nasal NO (nNO), electron microscopy (EM) and high-speed video microscopy analysis (HSVA) were assessed. Results were compared by laterality status and the period of diagnosis (before or after the 2009 consensus statement from the European Respiratory Society [32]). Patients with laterality defects had fewer tests performed than patients with situs solitus. The use of nNO (73% vs 62%) and HSVM (81% vs 71%) was lower in patients with laterality defects, while EM testing (82% vs 83%) stayed the same. This suggests that in patients with laterality defects are more often directly tested with EM. Overall, there was poor adherence to the 2009 consensus recommendations, with heterogeneity in diagnostic testing between countries.

Hannah Mitchison (UK) reported the prevalence of laterality defects and discussed the underlying clinical and genetic risk factors in a cohort of 389 PCD patients attending the management service in the UK.

Of all patients studied, 199 had a genetic diagnosis and 370 had a confirmed situs status through clinical imaging (surveying SS, SIT and SA). Out of the 370, 234 patients fulfilled the criteria required for having their organ defects classified and from this it was calculated that 17% had congenital heart defects (CHD), rising to 25% having CHD and/or organ defects other than SIT– much higher than previous estimates. Risk factors for CHD were found to include having a situs defect and having consanguineous parents.

A subset of nine genes (*RSPH1*, *RSPH4A*, *RSPH9*, *HYDIN*, *CCDC164*, *CCDC65*, *CCNO*, *MCIDAS*, *RPGR*) where mutations have not previously been associated with situs abnormalities in PCD patients was presented. A phenotypic continuum concerning PCD and CHD was suggested, as a pathogenic missense mutation in a known PCD gene (*DNAAF1*) was found to cause CHD with clinical evidence of PCD. Woolf Walker (UK) presented a study assessing body mass index (BMI) and bioimpedance spectroscopy (BIS) measurements in a cohort of 40 PCD patients under 16 years old attending University Hospital Southampton. Data on clinical phenotype, anthropometric measurements and nutritional intake of the patients were also collected. The study concluded that early nutritional intervention may have a positive impact on clinical outcome measures, and that monitoring of vitamin D may be important in PCD patients.

Andreia Pinto (Portugal) presented an overview of PCD diagnostics in Portugal and emphasized the need for further development in the Portuguese diagnostic network. She also highlighted progress made, especially in the identification of ultrastructural abnormalities in patients, and discussed the correlation between hallmark ultrastructural defects and ciliary beat pattern studied by HSVA.

Panayiotis K. Yiallouros (Cyprus) presented the pulmonary lobectomy as a clinical intervention using data from 2855 patients in the iPCD cohort. 163 lobectomized patients from 14 European PCD diagnostic centres were identified. The prevalence of lobectomy was higher in adult than in paediatric patients. In a nested case control study, lobectomized patients had worse lung function and steeper decline of forced vital capacity (FVC) with age compared to other patients in the iPCD cohort. They also reported that female gender and multiple lobectomies were associated with poorer prognosis and long-term outcomes. Participants discussed that it was not possible to know whether the patients who underwent lobectomy had worse lung disease than those who had not, and therefore whether the surgery itself was responsible for poor outcome.

Farheen Daudvohra (UK) presented a study on the relationship between the genetic variants and structural phenotype of outer dynein arms (ODA) using tomography to produce high-resolution 3D models of ciliary axonemal microtubular doublets and ODA volume ratios. They identified mutations associated with ODA structure in 39% of a cohort of 195 patients genotyped using NGS, in known PCD genes and a candidate gene; *DNAH9*. In patients with mutations in *DNAH9*, they found a significant deficiency in the ODA volume at the distal aspect when compared to the proximal aspect of the axoneme reflecting the DNAH9 protein positioned in the distal part of the cilia. It was concluded that use of 3D electron tomography can detect subtle changes in the ultrastructure of ODA in PCD patients.

### Ongoing and new BEAT-PCD projects

Several ongoing BEAT-PCD projects are in different stages of development or implementation, and an update on each was presented by the lead in each project. New members at the conference were encouraged to join and participate in these projects to widen the network of international collaborations.

Myrofora Goutaki (Switzerland) discussed the development of a disease-specific proforma as a tool for standardizing longitudinal data collection and follow-up care for patients with PCD. Recording clinical signs and symptoms in PCD is highly subjective and there is a wide variability in definitions and assessment [4]. The standardized proforma was developed through an adapted Delphi approach and was designed to capture information at different stages of the disease. It includes basic medical information, physical examination of lungs and heart, ENT examination, growth measurements, lung function measurements and other clinical tests, hospitalisations and treatment, interim medical history and information regarding lifestyle and environment. It is expected to improve the quality of collected data for better clinical care as well as prospective research. In addition it will allow easier communication and data exchange in a standardized way between collaborating centres. The next step is to pilot the proforma in both paediatric and adult populations, and to incorporate it in the database used for the iPCD cohort.

Bruna Rubbo (UK) presented an update on the PROVALF-PCD study (Prospective Observational multicentre study on Variability of Lung Function in stable PCD patients). The study is using spirometry measures to monitor the natural intra-individual and inter-individual variability of FEV_1_ z-scores in stable PCD patients. In addition, the variability of spirometry measures before and after pulmonary exacerbations will be evaluated.

Recruitment for the study is ongoing, and will end in April 2018. Seven centres are already active, and 91 patients have already been recruited. Patients will be followed up until May 2019 and spirometry measurements will be recorded at each clinic visit every 3-6 months, using a study-specific database.

Lynne Schofield (UK) presented an update on the international PCD physiotherapy network (IPCDPN). The IPCDPN aims to develop evidence-based practices and further collaborations for an international physiotherapy consensus document. A European survey has already been conducted to identify physiotherapists advising PCD patients on airway clearance. As a result, a physiotherapy network was set up in Basecamp, and teleconferences have been held to discuss future projects.

A manuscript on standard of care for physiotherapists, discussing the English national physiotherapy guidelines for children with PCD and covering areas such as service standards, airway clearance techniques, mucolytics and muco-active agents, exercise, and sinonasal management is in press.

### ERN-Lung PCD CORE

Heymut Omran (Germany) presented the structure and highlighted the benefits of being part of the EU-funded ERNs (European Reference Networks) for rare complex diseases; with emphasis on the PCD CORE of the ERN for Respiratory Diseases (ERN-Lung). These networks aim to improve patient care and epidemiological knowledge of rare diseases through creating centralized and highly specialised care centres, Europe wide collection of data, sharing of agreed clinical standards, and the development of strong collaborations between the ERN cores.

The integral role of the PCD registry was highlighted, which is a continuation of the PCD Registry set up during the 7^th^ Framework Programme (FP7) funded research project BESTCILIA [12]. This allows data to be stored centrally and utilised in different research projects. Proposed projects include: exploration of genotype-phenotype correlations; correlation of nNO production rates in molecularly defined PCD individuals; correlation of lung function parameters with other ERN-Lung Cores; variation of lung function parameters among siblings; exploration of microbiological results in PCD cohort; and a new consensus group for IF microscopy.

Healthcare providers applying for either full or supporting membership for the PCD CORE must meet the minimal requirements, which include the availability of key diagnostic tests and specialised treatments. Recruitment of new EU healthcare providers for patients with PCD is still needed and he emphasised that it will be easier for institutions affiliated with ERN to apply for EU funding, including ERN-specific funding opportunities.

### Revisiting BESTCILIA

BESTCILIA was an FP7 funded research programme which preceded BEAT-PCD.

Claudia Kuehni (Switzerland) presented the results of the observational trials in the iPCD cohort, which is now a BEAT-PCD adopted study and currently has 3507 PCD patients in 20 countries [11]. In 2017, the methods and first results of the iPCD cohort were published in the European Respiratory Journal [11], alongside a paper on growth and nutritional status and their association with lung function [33]. Further work using the iPCD cohort dataset is underway. During the conference, Florian Halbeisen (Switzerland) reported on lung function in PCD patients, and Panayiotis Kouis (Cyprus) presented a nested study in 20 centres on lobectomies from the iPCD cohort. Myrofora Goutaki (Switzerland) is investigating neonatal manifestations, and Florian Halbeisen (Switzerland) is evaluating the evolution of diagnostic testing of PCD in Europe. Planned studies will be focused on the clinical picture and national history of PCD, phenotypes, and the creation of a simple disease classification model. Data on symptoms, morphology and genetics will be added to the iPCD cohort to be used for future publications. The standardised PCD proforma will be linked to the iPCD cohort database and will provide further prospective clinical data on patients from centres that use it. The iPCD is a dynamic cohort – it is still possible for new centres to join, for participating to add more patients, and to add follow-up data on patients already registered.

Heymut Omran (Germany) presented the international PCD registry which is now part of the ERN-LUNG network [12]. The ERN-LUNG network is based on the exchange of expertise and clinical data on patient cases through the network with specific technical solutions. One of these technical solutions is the Data Warehouse for safe data storage and data analysis. Patients can register themselves in the international PCD registry for eligibility checks to participate in clinical trials. Both European and non-European countries can join this network as full or supporting members, respectively. The international PCD registry can help to improve highly specialised patient-centred cross-border care and can be linked with other ERN-LUNG Cores, including ERN-LUNG networks for cystic fibrosis (CF) and for non-CF bronchiectasis.

Panayiotis Kouis (Cyprus) presented an update on standardized diagnostic PCD testing in European countries with limited health care resources. A 24-month patient recruitment cycle in Cyprus, Greece, and Poland was initiated for diagnostic testing. Training workshops took place in Muenster, Athens, Krakow, and Limassol. Publications from this work package have included a meta-analysis dedicated towards diagnostic accuracy of nasal nitric oxide for establishing diagnosis of PCD [34]; a systematic review and meta-analysis on prevalence of PCD in consecutive referrals of suspect cases and the TEM detection rate [35]; and an international cross-sectional study on PCD [36]. Currently, a manuscript describing the development of an evidence-based diagnostic algorithm for PCD work-up is in preparation.

Jane Lucas (UK) presented updates on the development, validation, and translation of measures to assess Quality of Life in patients with PCD (QOL-PCD). These age-specific questionnaires were first developed in English and then translated into German, Dutch and Danish with cross-culturally validation. The questionnaires have now been translated into 10 languages. QOL-PCD was developed for children, teenagers, and adults with PCD, and for parents of children with PCD [17, 18] and then underwent psychometric validation [16]. The QOL-PCD questionnaires are free to use after the copyright holders have approved. Future actions of the QOL project includes a grant application for Parental Impact questionnaires for children aged <6 years.

Helene Kobbernagel (Denmark) presented the progress of the first multinational Randomized Controlled Trial (RCT) on Azithromycin in PCD. This double-blind, parallel group study examined the efficacy and safety of azithromycin maintenance therapy for 6 months in subjects with PCD. The primary outcome of this RCT is pulmonary exacerbations in patients with PCD. A total of 90 subjects were included from Rigshospitalet in Denmark, Copenhagen, VU Medical Center Amsterdam, Netherlands, University Hospital Muenster, Germany, Royal Brompton Hospital and University Hospital Southampton, United Kingdom, and Inselspital Bern, Switzerland. Data are being cleaned, processed, and locked in an electronic case report form (CRF) for further statistical analyses and unblinding of treatment allocation. The study protocol, rationale and recruitment of the Azithromycin trial has been published [14]. Several manuscripts are planned including assessing safety and efficacy of azithromycin maintenance therapy in PCD as well as associations between QOL-PCD outcomes and clinical measures such as spirometric values and lung clearance index derived from multiple breath washout.

### Training School Workshops

#### Transition for patients with PCD

Amanda Harris (UK) and Amanda Friend (UK) led a workshop that explored the concept of patient transition between paediatric and adult care using the “Ready Steady Go” Programme [37]. Data from UK centres show that patients with PCD have a higher adherence to treatment and better outcomes when they are appropriately transitioned to adult care. The Ready Steady Go Programme offers both tick-box proformas to make sure that healthcare professionals cover every necessary point and a more flexible approach, allowing individually based communication. The programme provides a platform for open exchange between patients and healthcare professionals by providing the time and opportunity to explore concerns that the patient might otherwise not feel comfortable to express.

#### Pre-clinical models for investigating PCD

Susana Lopes (Portugal), Dominic Norris (UK) and Monica Bettencourt-Dias (Portugal) led a workshop on pre-clinical models. The workshop was focussed on informing participants about range of model organisms suitable for use in PCD research. Different models that exhibit motile cilia and can be used to investigate PCD were discussed. Unicellular organisms included Paramecium, Chlamydomonas and Trypanosoma, whilst Planaria and Drosophila were suggested as invertebrate models. Zebrafish, Xenopus and mouse were given as examples of vertebrate models. The strengths and weaknesses of each organism as a PCD model were highlighted, and how different research objectives and experimental design influence the choice of model organism that a researcher might make. Researchers were advised to consider both the aims of their research and practical constraints such as time, budgetary requirements and skill set required for different model organisms when evaluating which could best aid their work.

#### Physiotherapy – education, techniques and tailoring a programme for patients with PCD

Paulo Buonpensiero (Italy) and Lue Philipsen (Denmark) facilitated a workshop on physiotherapy, focusing on key aspects of physiotherapy for PCD patients. The aim of this workshop was to highlight the many considerations needed to develop an effective and individualized physiotherapy regimen. The physiological principles of different techniques were explained. Important points for the selection of physiotherapy technique included: underlying pathology; the physiology of the airways; age of the patient; patient preference of technique; severity of disease; and level of compliance of the patient. Finally they discussed how clinicians can improve the effectiveness and efficiency of airways clearance techniques by ensuring that the airways are in their optimal physical state prior to doing physiotherapy. For instance, the use of mucolytics, a good inhalation technique and an effective inhalation device will improve the physical state of the airways by improving their hydration, reversing any bronchoconstriction and reducing irritation of the airways. Therefore, these preparation techniques could assist secretion clearance.

#### Nasal brushing techniques and how to process samples for the diagnostic pathway

Sarah Ollosson (UK), Amelia Shoemark (UK), Claire Jackson (UK) and Rob Hirst (UK) led a workshop on nasal brushing techniques and the diagnostic pathway. They educated the participants on how to take a good nasal brushing biopsy and recognise an adequate sample by HSVA. The main focuses of the practical session were how to prepare the media and brushes, how to prepare and position the patient and contraindications of the test. The talk summarised: how to prepare samples for HSVA, cell culture, immunofluorescence and fixation for TEM; how to recognize features of an adequate sample by light microscopy; and factors that may affect sample quality.

#### Laterality in PCD

This workshop was facilitated by Leonore Saúde (Spain), Susana Lopes (Portugal) and Dominic Norris (UK). The underlying mechanisms controlling left-right patterning were discussed, enabling a detailed and in-depth examination at the evolutionary conservation and divergence of laterality patterning. Group discussion of the nature of laterality defects observed in PCD patients followed, with situs randomisation being of particular interest. The facilitators emphasised that ciliary motility defects at the embryonic node lead to randomisation of situs, which explains the range of laterality defects observed in PCD patients.

#### Future prospects for cross-sectional imaging in PCD: can we build on conventional CT and is there a role for MRI?

The workshop was led by Tom Semple (UK). The first part of the workshop focused on the fundamentals of conventional imaging and included an interactive discussion about the principles of chest x-ray and computed tomography (CT). In the second part of the workshop, structural magnetic resonance imaging (MRI) was discussed, including comparisons on the different characteristics and uses of MRI and CT. Scoring sheets have limitations as they are time consuming, have significant inter-reader variability, require training and depend on sub-scores and reporting of composite scores. Quantitative imaging techniques were also discussed, including semi-quantitative visual scoring and mapping techniques for both CT and MRI. Currently MRI of the lung remains a research tool and there are no disease-specific scoring systems for CT; however, imaging is an active area of research at many centres and it is hoped that standardised approaches for imaging the PCD lung will evolve in the near future.

#### Nasal nitric oxide in the diagnostic workup of PCD

The workshop was facilitated by Jane Lucas (UK), Claudia Kuehni (Switzerland), Bruna Rubbo (UK) and Amanda Harris (UK). Claudia discussed how to identify patients for diagnostic testing. The positive predictive value of nNO measurements within a specialised PCD diagnostic centre is 44% but this falls to 0.6% if measurements are taken with general pulmonology settings where the rate of false positives will be proportionately much higher. The PICADAR tool [38] and Leigh score [39] for clinical diagnosis of PCD were also discussed, as were the ERS Task Force recommendations of diagnostic nNO use [40]. A series of case vignettes facilitated discussion of the advantages and disadvantages of each diagnostic test, as well as the recommendations of which procedure is to be used for nNO measurement according to patient age. The workshop finished with hands-on nNO measurement experience.

#### Clinical trials in PCD

The workshop was facilitated by Kim G Nielsen (Denmark) and Helene Kobbernagel (Denmark). The aims of PCD treatment and a brief examination of current clinical non-evidence based options were discussed. The facilitators then gave an overview of current trials and summarised clinical outcome measures that have been or are being used in PCD. Participants analysed design of these trials, including exclusion and inclusion criteria. The necessity of including multiple centres in a PCD trial was discussed, as were the implications this may have for trial design. Participants were divided into two groups consisting of mixed scientific and clinical backgrounds for an interactive conversation on study designs, and then had the opportunity to design their own randomised clinical trial. One group considered a study concerning inhalation of dornase alfa and the other drafted a study on use of inhaled antibiotic for chronic pulmonary infection with Pseudomonas Aeruginosa.

#### Patient education following a new diagnosis of PCD

Gemma Marsh (UK), Laura Baynton (UK) and Fiona Copeland (UK) facilitated a workshop focusing post-diagnosis patient education. The facilitators presented an overview of the patient education programme at the Royal Brompton Hospital, London. The discussion revolved around explaining PCD to a newly diagnosed patient or parent and the impact the diagnosis might have in their lives, as well as patient management of their condition and the importance of complying with treatment. The facilitators gave practical tips and presented examples of patient case studies to help participants to understand the patient’s perspective on receiving a PCD diagnosis. Fiona Copeland shared her own experiences as a parent of two children with PCD.

### Consensus Statement Workshops

#### Transmission electron microscopy: hallmark defects

European diagnostic guidelines recommend ‘a hallmark defect’ in TEM as a confirmatory diagnostic test for PCD [41]. However, there is currently no international classification of ultrastructure for cilia biopsies and there is considerable heterogeneity among pathology reports describing PCD. Thirteen invited attendees representing nine countries met for the second time to develop a consensus statement for reporting ciliary ultrastructure by TEM for PCD diagnosis. Aims for the consensus guidelines include defining hallmark defects diagnostic for PCD; defining what should be included in a TEM report to assist multidisciplinary diagnosis of PCD; and defining adequacy of a diagnostic sample.

During the workshop experts discussed the results to date and the draft manuscript. The group planned a final Delphi survey for two items on which 80% consensus had not yet been reached and drew up a strategy to test the guideline before publication. A final face to face meeting is planned at the next BEAT-PCD conference.

#### Infection control

The development of a consensus statement for PCD and cross infections was initiated at the previous BEAT-PCD Training School and Conference [9] at a workshop chaired by Helle Krogh Johansen (Denmark) and Kim G Nielsen (Denmark).

An e-survey was circulated to the group prior to the meeting raising questions regarding infection prevention and control in PCD, including microbiology measures and treatment strategies to prevent cross-infections in out-patient and in-patient settings. Participants discussed the survey responses and concluded that additional surveys need to be taken before a formal agreement can be achieved.

#### Defining pulmonary exacerbations

The inaugural BEAT-PCD Conference in 2015 identified the need for a consensus statement to define pulmonary exacerbations in children and adults with PCD as an outcome measure for clinical trials. Paediatric and adult pulmonologists, along with a nurse, physiotherapist and patient representatives have completed three rounds of e-Delphi surveys since the BEAT-PCD Conference in 2017 to develop the definition. The results of these surveys were discussed at this conference, with fine-tuning of the wording of some of the items. It concluded that one or two further e-surveys will be needed before the final definition is agreed and presented at the next BEAT-PCD conference.

### Short Term Scientific Missions

Short term scientific missions (STSMs) provide an opportunity for early stage researchers (ESRs) to visit a research institution in another country, with the aims of sharing knowledge and skills between centres. This is intended to advance PCD research at their institution and to develop collaborative projects. Developing PCD centres can also use STSMs to invite an expert to provide on-site training and expertise. Up to fifteen bursaries are awarded each year. More information on STSM applications can be found on the BEAT-PCD website (http://www.beatpcd.org/).

During this Conference and Training School, STSM participants presented overviews of their experience during their visit.

Nuria Camats (University Hospital Vall d'Hebron, Barcelona) visited Heymut Omran in Münster, Germany. During her 2-week stay, she gained experience on how to perform genetic analysis of PCD-related genes. She also received hands-on training regarding IF microscopy of ciliated respiratory epithelial cells. She plans to apply these new skills to improve the accuracy of diagnostics and to develop candidate-variant functional studies in her home institute.

Margarita Kaliva (University of Cyprus) also visited Heymut Omran in Münster. She gained experience and training on how to establish an ALI- culture system, which she will implement in her home institute.

Joe Hayes (University of Leicester, UK) was invited as an expert to visit the University Hospital Vall d'Hebron, Barcelona, and was hosted by Antonio Moreno Gald. During his visit, he shared his experience of IF in PCD diagnosis using a panel of ciliary protein markers (DNAH5, DNALI1, RSPH4A and GAS8), and on cell culture including ALI technique. In Barcelona he performed IF microscopy on PCD patients and healthy controls and assisted the host centre in establishing ALI-culture.

Loretta Müller (University of Bern, Switzerland) was hosted by Jane Lucas at the PCD diagnostic centre in Southampton UK. More specifically, she was introduced to IF and ALI techniques as well as training in HSVA. She received an overview of the organization and structure of the PCD diagnostic centre, and aims to establish a comprehensive diagnostic service at her home centre.

Pierrick le Borgne (University Paris-Saclay, France) visited Hannah Mitchison at University College London, UK. Through a collaboration with Mahmoud Fassad (UK), he performed RNAi gene silencing of *C11orf70* in Paramecium, and analysed Paramecium swimming velocity, ciliary beat frequency and pattern, and ultrastructure by TEM.

### Poster session

The BEAT-PCD Conference and Training School included a poster session. Titles and authors are given in Table 1, with permission. Abstracts are presented in Part 2 [link].

**Table 1:** Poster titles presented by authors (country of first author) at the 3rd BEAT-PCD Conference

Poster title	Authors (*country of first author*)
Is there a defect in ENaC activity in the nasal epithelium of patients with PCD?	Harman K, Alton EWFW, Davies JC, Waller MD, Crowley S (*UK*)
Ciliary functional analysis using videomicroscopy: time for a standardisation	Kempeneers C, Seaton C, Espinosa BG, Chilvers (*Belgium*)
Loss-of-function mutations in PIHD3 cause X-linked PCD with outer and inner dynein arm defects	Hoben I, Paff T, Loges NT, Aprea I, Wu K, Bakey Z, Haarman EG, Daniels JMA, Sistemaans EA, Bogunovic N, Dougherty GW, Große-Onnebrink J, Matter A, Olbrich H, Werner C, Pals G, Schmidts M, Omran H, Micha D (*Germany*)
Increased plasma ceramide and sphingomyelin levels in the plasma of PCD patients	Topcu DB, Tugcu G, Ozcan F, Aslan M, Esref S, Hizal M, Yalcin E, Ersoz D, Ozcelik U, Kiper N, Lay I, Oztas Y (*Turkey*)
The role of laterality signals from the left-right organiser in zebrafish gut patterning	Bota C, Lopes S (*Portugal*)
Downstream target of Pkd2 affects nodal signalling regulator in left-right development.	Jacinto R, Lopes S (*Portugal*)
ENKUR- a novel heterotaxy gene	Menchen T, Sigg MA, Lee C, Jungnickel MK, Dougherty GW, Pennekamp P, Florman HM, Wallingford JB, Reiter JF, Omran H (*Germany*)
Successful pregnancies for six women with PCD	Sivaramakrishnan H, Cottee A, Coon C, Morgan L (*Australia*)
Two siblings with PCD and hepatic involvement	Hizal MG, Bilgic E, Taskiran E, Atilla P, Akcoren Z, Gunaydin O, Ozen H, Esref S, Emiralioǧlu N, Yalcin E, Ersoz D, Kiper N, Yuce A, Ozcelik U (*Turkey*)
Homozygous loss-of-function mutations in MNS1 cause laterality defects and male infertility	Hjeij R, Ta-Shma A, Perles Z, Dougherty GW, Abu Zahira I, Letteboer SJF, Antony D, Darwish A, Mans DA, Spittler S, Edelbusch C, Cindric S, Menchen T, Olbrich H, Stuhlmann F, Aprea I, Pennekamp P, Loges NT, Breuer O, Shaag A, Rein AJJT, Gulec EY, Gezdirci A, Abitbul R, Elias N, Amirav I, Schmidts M, Roepman R, Elpeleg O, Omran H (*Germany*)
Creation of a Danio rerio mutant using CRISPR-Cas9 as a model system to study PCD.	Rasteiro M, Lopes S (*Portugal*)
Continence assessment in paediatric patients with PCD	Wilkins H, Friend A, Harris A, Keenan V (*UK*)
A new possible role of V-ATPase in the left-right development	Pestana S, Lopes S (*Portugal*)
Immunofluorescence is a useful adjunct to TEM for diagnosis of PCD	Canoy I,MacKenney K, Clarke C, Morgan L, Buddle L, Hughes L (*Australia*)
A four-year experience of a PCD diagnostic centre in Greece	Chatzipirasidis G, Douros K, Mpoutopoulou B, Papadopoulos M, Grammeniatis V, Dimakou K, Priftis KN (*Greece*)

### Difficult case management discussions

The management of PCD patients remains challenging, with limited evidence base to guide clinicians on best practice [1, 42]. Discussion of difficult clinical cases is therefore key to further developing expert consensus on management. Five such challenging cases were presented during this interactive session.

Cases presented included:

Eric Haarman (Netherlands) presented a 9 year old boy with bi-allelic *DNAH5* mutations, who has had significant clinical decline, with seasonal variation. Bronchoscopy revealed copious sticky mucus but culture of the bronchial lavage was negative. He received hypertonic saline, maintenance oral and regular intravenous antibiotics. The audience suggested further investigations for infection specifically for nontuberculous mycobacteria by use of selective media and for fungal infection, including investigating for allergic bronchopulmonary aspergillosis, and also considering co-morbidities such as asthma. The audience advised continuing aggressive management with close follow-up.

Nisreen Rumman (Palestine) presented a case of siblings from a consanguineous marriage with bronchiectasis, clubbing, hepatosplenomegaly and leucopenia. PCD diagnostic workup was normal, as were metabolic/haematological and enzymatic investigations. Whole exome sequencing revealed a mutation in *STXBP2,* which confirmed Familial Hemophagocytic Lymphohistiocytosis. The siblings were referred for bone marrow transplant. This highlights the importance of seeking alternative diagnoses to PCD, as therapies differ and may be curative.

Julie Duncan (UK) presented three cases from the Royal Brompton Hospital:

- Case 1 had PCD phenotype with respiratory symptoms and dextrocardia, low nNO and cilia beating at the tip but static at the base. TEM and IF were normal (genetics pending). The patient developed seizures and was diagnosed with neurofibromatosis (NF) type 1. A possible association between NF and PCD, or whether this was two diseases in one patient, was discussed. No one else in the audience had experience of a co-existence of these two conditions.
- Case 2 was siblings demonstrating a severe phenotype, with stiff dyskinetic ciliary beating by HSVA and GAS8 was absent by IF. Genetics revealed mutations in *CCDC164*. The audience agreed that these patients suffered from a more severe disease progression. Possible predictors of lung function decline were discussed. The importance of genotype/phenotype association studies was raised and agreed upon by the audience.
- Case 3: A patient with PCD and persistent right middle lobe consolidation requiring bronchoscopy had developed a pneumothorax after the procedure. The group agreed that pneumothorax is not expected to be more common in patients with PCD compared to other disease groups.

### Difficult diagnostic cases

Despite recent evidence-based guidelines for the diagnosis of PCD, many cases remain inconclusive [40, 41, 43]. Accurate diagnosis of subtle cases requires experience, and yet PCD diagnosis is not always clear even in reference centres. Discussion of complicated cases across centres is therefore important.

Carolina Constant (Portugal) presented two cases with inconclusive diagnoses:

Case 1: An 8 year old, who was born at term and admitted to NICU due to aspiration pneumonia, presented with ongoing respiratory symptoms, and left lower lobe collapse leading to a lobectomy. PCD diagnostics revealed a PICADAR score of 6 [38], low nNO, clusters of cells on TEM but no cilia observed, minor microtubule abnormalities with many inflammatory cells giving the impression of infection. The audience advised to repeat TEM, lack of cilia commonly being a secondary problem caused by infection. Mutations in *CCNO* or *MCIDAS* can be primary causes of reduced motile cilia.

Case 2: A boy, born preterm at 33 weeks of gestation, developed bronchiolitis obliterans following adenoviral infection at 14 months of age. Now 12 years old, he presented with ongoing respiratory symptoms and ear infections, clubbing, reduced lung function and diffuse bilateral bronchiectasis on CT with small airways involvement. PCD work-up demonstrated a PICADAR score of 6 [38], normal nNO, TEM with some cilia with partial ODA defects, and HSVA revealing normal CBF but with stiff movement. The audience advised a repeat of TEM and to perform IF.

Claire Jackson (UK) and Rob Hirst (UK) presented cases of patients with *CCDC103* mutations. Both patients had a strong clinical history of PCD, one with SI. The PCD diagnostics for the first case revealed abnormal nNO, most IDAs missing, normal CBF and subtle CBP abnormalities. The second case had normal CBF, 20% dyskinetic and normal TEM. These cases highlight the importance of genetics to help establish a diagnosis – especially if there is a strong clinical suspicion when other investigations are inconclusive [44].

Rob Hirst (UK) presented a 9 year old African boy with severe respiratory disease from 12 months, ear infections, atopy and clubbing. PCD work-up demonstrated dyskinetic cilia, normal CBF with whip-like motion occasionally, long cilia and expanded sections of cilia shafts (also after culture). TEM revealed long “cobra head” cilia with a normal axoneme structure. The patient died of pneumonia before work-up was completed. The group concluded that he likely had a ciliopathy. The audience was not aware of “cobra-head” cilia previously reported in the literature.

Louise Hughes (Australia) and Lucy Morgan (Australia) presented a patient with a strong PCD clinical phenotype, low nNO, and reduced CBF (<5 Hz). TEM and IF were abnormal and revealed two subgroups of cilia. There was a clear deficiency of IDA in isolation (i.e. not occurring in combination with an ODA defect, or with microtubular disarray), despite abnormal staining with DNAH5 and GAS8 markers. The group agreed that there was a possibility of both defects co-existing.

Nicola Ullman (Italy) presented a 10 year old child born with oesophageal atresia type IIIC and dextrocardia, who suffered from chronic vomiting and respiratory symptoms post-operatively. A CT scan showed severe bronchiectasis with parenchymal destruction of right lower and middle lobes. PCD diagnostics showed only reduced cilia motility (6.3Hz) and genetics revealed two heterozygous mutations in two different genes, *DNAH8* and *DNAAF5*; both maternally inherited, and mother was clinically well. The audience discussed that *DNAH8* is not a respiratory ciliary gene and PCD diagnosis requires bi-allelic mutations in one gene [41]. The audience attributed the bronchiectasis to aspiration and that PCD was very unlikely.

Jane Lucas (UK) presented a case with normal nNO, abnormal TEM showing some cilia with absent central apparatus, others with transposition defects and others with normal ultrastructure. A second TEM sample was entirely normal. HSVA demonstrated rotating cilia on that occasion. Genetics revealed a mutation in *RSPH1* [45–47]. This case demonstrated that non-hallmark cilia ultrastructure defects can change. This also serves as an example of a PCD case with nNO within the normal range.

### Early stage researchers networking forum

The 1^st^ Early Stage Researchers (ESR) networking forum was held during the BEAT-PCD Training School, led by ESR representatives Myrofora Goutaki (Switzerland) and Bruna Rubbo (UK). Forty-four participants attended the event, including postgraduate students and postdoctoral researchers, clinicians, and healthcare professionals. The ESR representatives highlighted several prospects for ESR to participate in the BEAT-PCD network activities. These include: developing the e-newsletter; organising the conference; chairing sessions; and writing the conference proceedings. STSMs were highlighted as a unique opportunity for ESR to learn new skills, develop joint projects and work collaboratively with other PCD groups. The application process was explained and participants heard from previous researchers who have completed STSMs.

Twenty-two participants completed a feedback survey (50% response rate). Respondents rated the networking forum 8 out 10 and all of those that completed the survey requested a 2^nd^ ESR networking forum at the next BEAT-PCD Training School. The majority of respondents expressed an interest in discussing the following topics at future ESR events: project design and management; writing a grant application; and small breakout groups to discuss collaborative projects.

### Evaluation feedback from participants

**Fig. 2:**
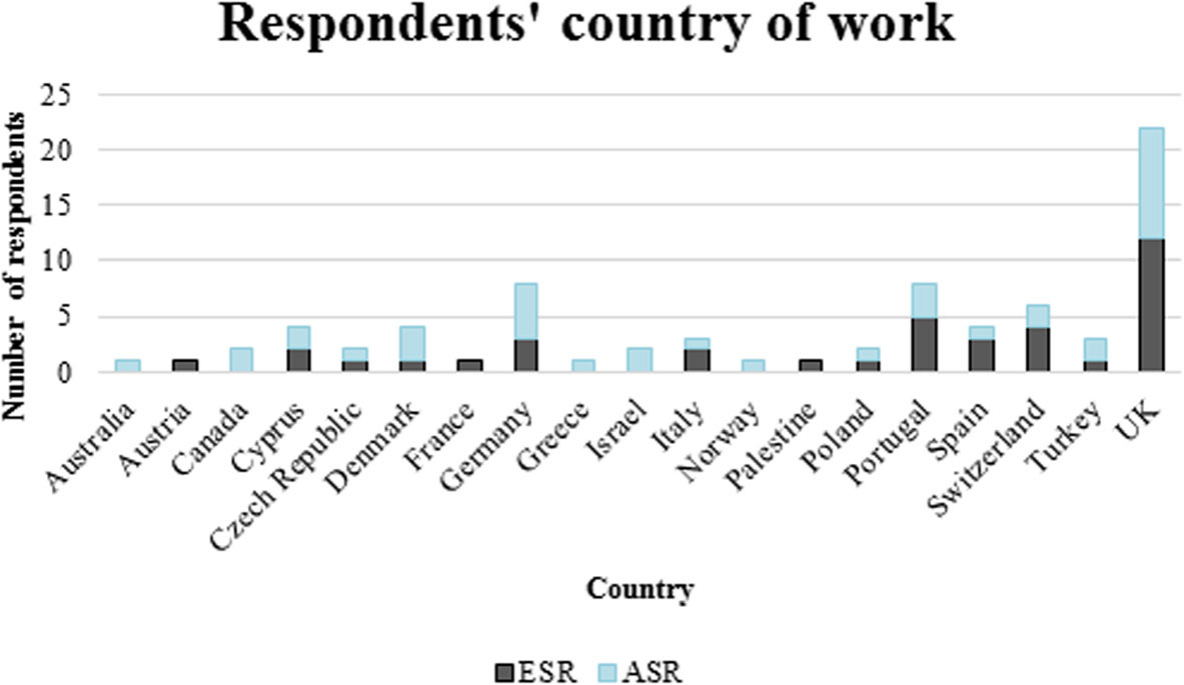
Country of work of respondents of the feedback survey for the 3^rd^ BEAT-PCD Conference & 4^th^ Training School. ESR: Early stage researcher, ASR: advanced stage researcher

An online feedback survey was circulated to participants following the Conference and Training School. Seventy-six respondents (61% response rate) from 19 countries completed the survey (Fig. 2). Half of the respondents were ESR, of which 53% had attended previous BEAT-PCD Conferences [9].

**Fig. 3:**
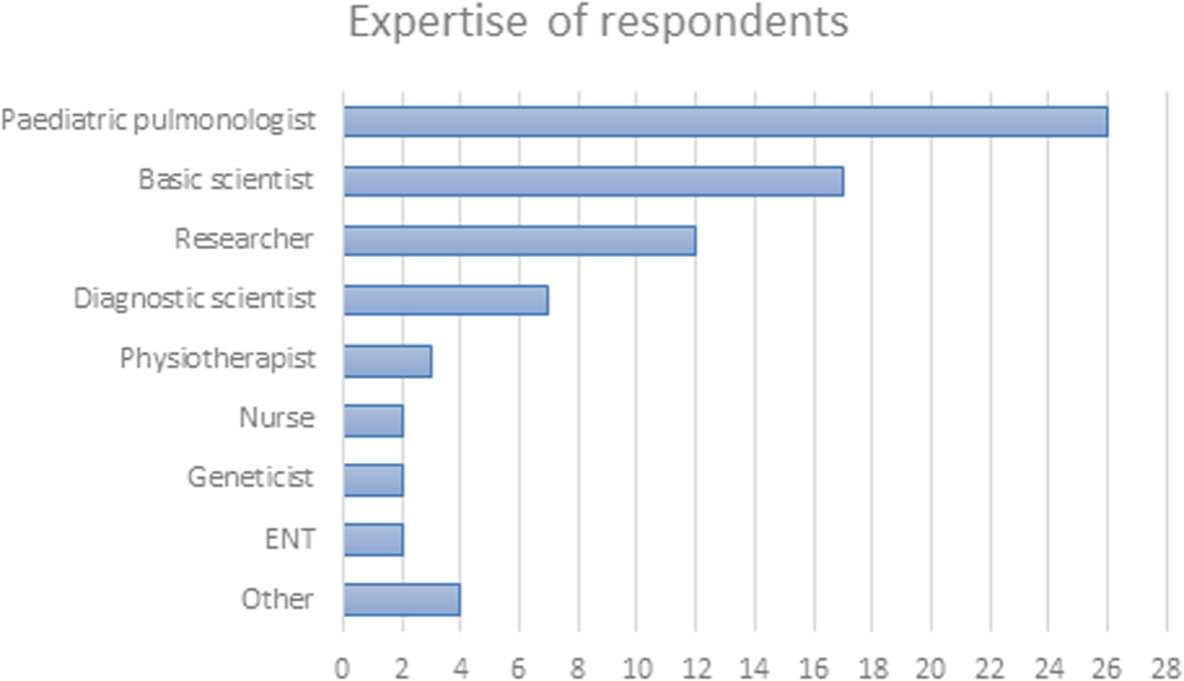
Main expertise of respondents of the feedback survey for the 3^rd^ BEAT-PCD Conference & 4^th^ Training School. ENT: ear, nose and throat specialist. The term “Researcher” includes epidemiologists; and “Other” includes pathologists, radiologists, microbiologists and patient representatives

Participants highlighted the opportunity to network with experts from varied backgrounds (Fig. 3) and PCD research groups as one of the best features of the Conference, along with the plenary talks, oral presentations and difficult case discussions. A third of ESR stressed that the Training School interactive workshops were their preferred activity.

Both ESR and Advanced Stage Researchers (ASR) rated the event 9 out of 10, with 92% of respondents’ stating that the Conference and Training School met their expectations and addressed their needs.

### Summary

The BEAT-PCD Conference and Training school brought together clinical specialists (paediatricians and adult pulmonologists, ENT, physiotherapists, specialist nurses) and scientists from varied backgrounds (PCD diagnostics, genetics, imaging, cell biology, microbiology, bioinformatics). This multidisciplinary conference continues to provide an interactive platform for clinicians and researchers from andpromotes the exchange of ideas through a programme of lectures, poster sessions, workshops and discussions. The next BEAT-PCD conference and training school will be held in Poland in 2019.

## Abbreviations

ALI: Air liquid interface
ASR: Advanced stage researcher
BEAT-PCD: Better experimental approaches to treat PCD
CBF: Ciliary beat frequency
CBP: Ciliary beat pattern
CF: Cystic fibrosis
CHD: Congenital heart disease
CT: Computed tomography
ENT: Ear, nose and throat
ESR: Early stage researcher
FEV_1_: Forced expiratory volume in 1 second
HSVA: High speed videomicroscopy analysis
IDA: Inner dynein arm
IF: Immunofluorescence
iPCD: International PCD (Registry)
IPCDPN: International PCD physiotherapy network
MRI-: Magnetic resonance imaging
NGS: Next generation sequencing
nNO: Nasal nitric oxide
ODA: Outer dynein arm
PCD: Primary ciliary dyskinesia
PICADAR: PrImary CiliARy DyskinesiA Rule
QOL: Quality of life
RCT: Randomised controlled trial
SA: Situs ambiguous
SIM: Structured illumination microscopy
SI(T): Situs inversus( totalis)
SRM: Super-resolution microscopy
STORM: Stochastic optical reconstruction microscopy
STSM: Short term scientific mission
SS: Situs solitus
(T)EM: (Transmission) electron microscopy
